# Design and analysis of multi-layer silicon nanoparticle solar cells

**DOI:** 10.1038/s41598-022-17677-z

**Published:** 2022-08-02

**Authors:** Sayyed Reza Mirnaziry, Mohammad Ali Shameli, Leila Yousefi

**Affiliations:** 1 Department of Electrical Engineering, University of Qom, Qom, 3716146611 Iran; 2 School of Electrical and Computer Engineering, College of Engineering, University of Tehran, Tehran, 1417614411 Iran

**Keywords:** Nanophotonics and plasmonics, Electrical and electronic engineering, Photovoltaics

## Abstract

We investigate the concept of nanoparticle-based solar cells composed of a silicon nanoparticle stack as a light trapping absorber for ultrathin photovoltaics. We study the potential of using these inherently nanotextured structures in enhancing the light absorption. For this, a detailed optical analysis is performed on dependency of the cell response to parameters such as the number of particle layers, lattice structure and angle of incidence; Optical response of these cells are then compared with the results in conventional silicon solar cells. Moreover, we propose various configurations to apply these submicron particles as a p–n junction solar cell. We also compute the electrical performance of selected configurations. In doing so, key issues including the effect of contact points between nanoparticles and impact of loss are addressed. In the end, we show how $\mathrm{SiO}_2$ nanoparticles on top of the cell structure can enhance the photocurrent. The appropriate range of $\mathrm{SiO}_2$ particle size is also obtained for the typical cell structures.

## Introduction

Ultrathin solar cells are referred to a group of photovoltaic structures possessing light absorbers with a thickness of at least an order of magnitude smaller than conventional solar cells^1^. These cells have drawn attentions for decreasing the raw material requirements, their flexibility and bendability^2,3^. Despite their reduced thickness, optical path length is improved by engineering the cell structure so as to compensate their low absorption. Ultrathin solar cells are expected to be fabricated with low-cost techniques via increased fabrication throughput^4^; for instance, they may be realized without protective glass layers^3^ or their active layer can be deposited with lower deposition techniques^1^. These cells can show robust performance in cell dislocations and low light-induced degradation^5,6^. Besides, bulk recombination mechanisms such as Auger recombination—are limited, which result in higher open-circuit voltages, and carrier collection is facilitated on contacts^1^. With these features, the concept of ultrathin photovoltaics has experienced a fascinating growth through the last decade and found applications in spacecrafts^3^ due to their short carrier diffusion lengths, which brings immunity against radiation damages. In addition, thanks to their flexibility, these cells are candidates to supply energy for portable devices in remote areas.

Attempts for design and realization of ultrathin solar cells have been concentrated on studying both the electrical and optical aspects; On the electrical side, common analysis include optimization of cell absorbers bandgap^7,8^, together with studying photo-carrier drift, diffusion, generation and recombination using carrier transport equations^9,10^. On the optical side, the absorption behavior of a cell is the key parameter that determines how efficient a cell architecture is in producing higher photocurrent. The main drawback against high cell efficiencies is insufficient light absorption in ultrathin structures. Due to this, researches on these cells are often directed toward finding light management architectures with practical values^6,11–14^. For instance, using proper anti-reflection coatings and embedding back mirrors^15,16^, using periodic nano-gratings on the front^17–19^, or random pyramids on the front and back of ultrathin silicon layers to achieve omnidirectional reflectance^20^. Optical confinement has also been explored through the excitation of edge states around the photonic topological insulator^21^. Considering these, the attempt has therefore been focused on configuring the structure both optically and electrically to preserve high short circuit currents while decreasing the thickness.

The efficiencies reported for ultrathin solar cells are also promising; in GaAs cells of thickness around 205 nm the conversion efficiency has reached 19.9%^3^. For CIGSe cells with the thickness of 1.2 $\upmu$m, the efficiency of 11.27% is reported in^22^. In ultrathin silicon solar cells, the efficiency of 8.6% is reported for a 1.1 $\upmu$m absorber, that although is lower than conventional cells, it shows a remarkable progress toward realizing a Lambertian model in ultrathin cells^1^.

Pattering an ultrathin structure can, however, complicates realization of these cells, and even contradicts with their principle benefits. Thus, simple cell configurations with reasonable efficiencies are most often preferred. Among various techniques to increase the short circuit current in ultrathin cells, using randomly roughened surfaces has shown promising results^6^. In this regard, the beneficial effect of nanoparticles on absorption enhancement and broadening solar spectral band for ever thinner solar cells has been extensively addressed^23–26^; In terms of fabrication, in contrast to photonic crystal patterns, nanoparticles can be made and deposited via lower cost techniques^27^. While attentions on ultrathin solar cells have been mainly drawn toward GaAs solar cells^28^, the low cost silicon solar cells of this type possess commercially more chance to be employed in widespread terrestrial applications with low energy requirements.

In this paper, we demonstrate multi-layer Silicon Nano-Particle (SNP) solar cells as a promising photon management technique in ultrathin photovoltaics. We show how this inherently textured architecture acts as a light absorber while having the potential to separate and transport photo-generated carriers. We compare the optical properties of a structure composed of these Mie scatterers with planar cells of the same thickness and provide a comprehensive analysis on the cell behavior for different number of particle layers when exposed to oblique incidence and also for various particle periodicity. Then, we study different scenarios to tailor the silicon nanoparticles as the active layer of a realizable cell. Next, we concentrate on an appropriate structure and optimize its geometrical and electrical parameters. In order to further improve the absorption, we examine the effect of distributing $\mathrm{SiO}_2$ nanoparticles on the cell front. Finally, we estimate the expected power conversion efficiency of the cell and compare it with the efficiencies reported in the literature.

## Electromagnetic properties of SNP absorbers with various parameters

**Figure 1:**
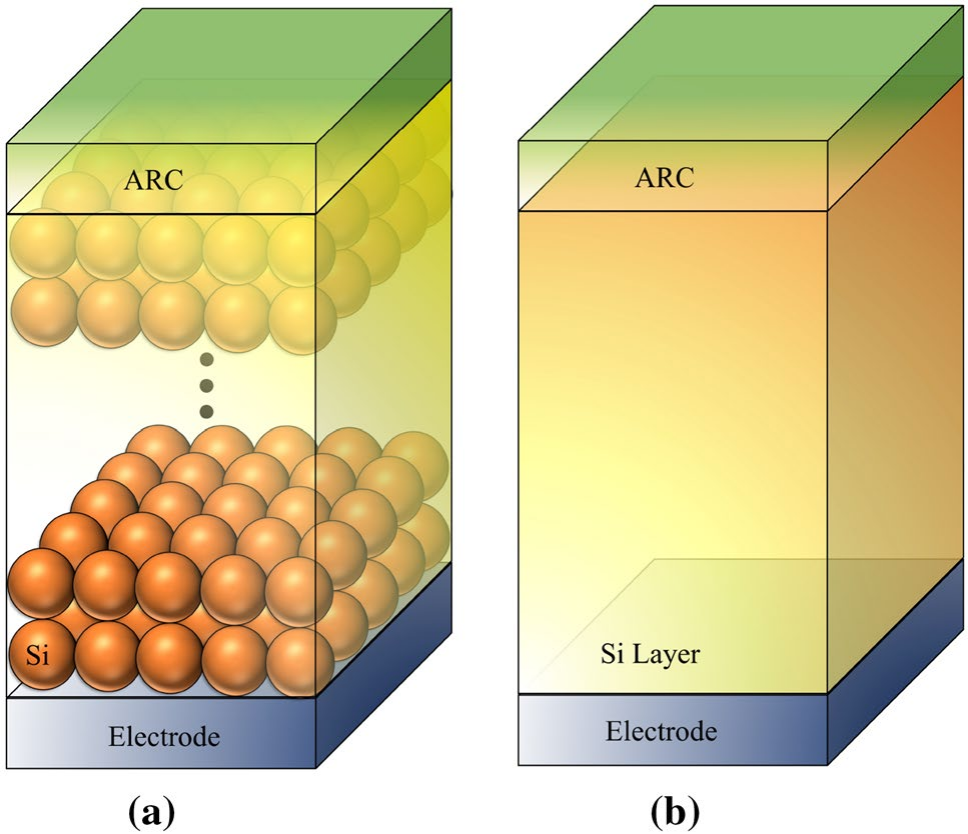
(**a**) The schematic of a structure composed of identical silicon nanoparticles and an ARC on the top. (**b**) A simple planar structure with silicon thickness identical to (**a**).

We here concentrate on the absorption properties of SNP layers and compare them with planar silicon layer. The two structures are shown in Fig. 1a,b. As shown in this figure, the silicon nanoparticles are densely stacked inside a dielectric medium. We also assume a metallic contact (silver) below, and an anti-reflection coating(ARC) above the absorbers to further resemble a real cell. We assume that particles have an identical size of the order of a few hundred nanometers; this dimension range ensures achieving a remarkable light trapping through the frequency spectrum that contributes in photo-generation (i.e. $\lambda$ = 300–1100  nm). Assuming the spherical shape of silicon nanoparticles, by reducing the particle radius, a fewer number of Mie resonances are excited and this leads to a lower light absorption. In addition, we will see that as the particle radius increases above 500 nm, the absorption enhancement—in comparison to a conventional cell—becomes negligible. This is because the absorption for the silicon nanospheres with a radius of 500 nm or larger will be close to the unity in the bandwidth of the solar cells. Light trapping in this particle-based structure is enhanced due to the excitation of whispering gallery modes. Moreover, from the ray optics viewpoint, the random path length of light beams inside the structure increases total absorption. With regard to the flat silicon layer shown in Fig. 1b, at $\lambda < 500\,\mathrm{nm}$, the absorption is due to the intrinsic loss of the crystalline silicon. In this range, the optical responses of both structures are very close together. At higher wavelengths, the Fabry–Perot resonance is the only mechanism for trapping light in the flat structure, and it happens only at limited number of wavelengths. In the following sections, we examine various aspects of distributed silicon nanoparticles in light absorption, if they are employed instead of the silicon layer.

### The number of NP layers

**Figure 2:**
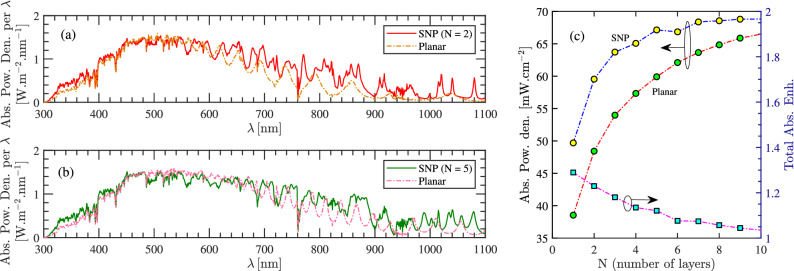
Comparison of an SNP and planar cell: the spectral absorbed power density per wavelength under one-sun illumination when (**a**) number of layers, N = 2 and (**b**) N = 5. (**c**) Total absorbed power density and enhancement as a function of N to compare an SNP and planar cell (NP radius = 300 nm).

We first study the impact of the number of particle layers in Fig. 1a on the absorption. The aim is to explore how many layer is beneficial in reaching higher absorption in comparison to a conventional cell in Fig. 1b. We assume that particles are spherical with a selected radius of 300 nm. Particles are assumed to be inside a carrier transport medium with the refractive index of 1.8, which is an average value for a number of materials such as PEDOT:PSS polymer and spiro-OMeTAD at the interested frequency spectrum of sunlight. The thickness of this medium above the upper layer is assumed to be 75 nm, which imitates the typical thickness of an ARC. Figure 2a,b show the absorption spectra of a multi-layer SNP absorber with two (N = 2) and five (N = 5) layers of silicon nanospheres when compared with that of a flat layer (by integrating the obtained graphs over the wavelength interval, we can compute the sunlight power density that is absorbed by the structure). As can be seen, a particle-based structure and a flat layer behave the same at short wavelengths in both figures. This was expected as the penetration depth is such small that the optical power is absorbed regardless of the considered configuration change. However, these particle-based structures provide improved absorption, at longer wavelengths. Moreover, by comparing the two figures, higher absorption is achieved when N = 5. Note that, if we assumed that the two structures (i.e. the multi-layer SNP and a flat structure) should have identical absorber volume, we would reach even further discrepancy in their absorption at long wavelengths.

Although the absorption is enhanced as the number of layers increases, the total absorption approaches that of a planar structure. This is shown in Fig. 2c where the total absorbed power density—at the interested wavelength interval—is computed for the particle-based structure and the planar one, as a function of the number of layers. We have also defined total absorption enhancement as the ratio between the total absorbed power density of the two structures. From the figure, increasing the number of SNP layers reduces the advantage of the SNP structure in comparison to a planar one. This indicates that the SNP cell is optically preferable only when a few layers are used.

### The lattice type

Particles in each layer of the structure shown in Fig. 1a may be arranged in different forms. Considering a dense distribution, a random arrangement for particles is a cheaper choice and practically preferred for mass production. Despite this, in terms of numerical analysis, one often has to consider a sort of periodicity to reduce the simulation domain. Knowing the impact of various particle distributions on the absorption behavior helps in finding an average expected response of a random distribution. Before studying the impact of particle arrangements, we emphasize that dense distribution of SNPs are much more desirable than sparse ones for solar cell applications. This is because SNPs are assumed to be the main absorber in the cell. Thus, any distance between them reduces the absorption of incident photons. As SNPs become closer, a stronger coupling will be formed between the optical fields inside the cell absorber.

**Figure 3:**
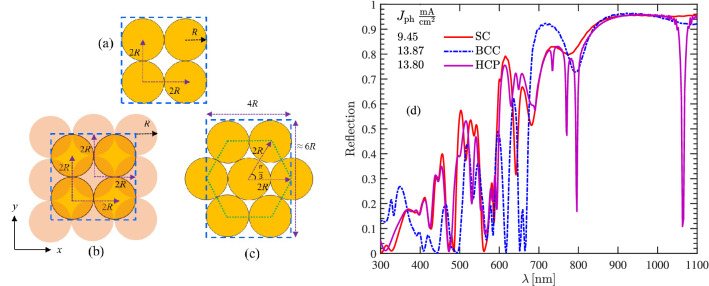
Three forms of lattice arrangement for the Si NPs multi-layer structures are shown in Fig. 1. The top view of (**a**) SC, (**b**) BCC, and (**c**) HCP. (**d**) Reflection of Si NPs with different arrangements which is coated by a dielectric layer (NP radius of 100 nm, the dielectric layer refractive index of 1.8, and thickness of 100 nm).

We restrict our study to a lattice composed of two layers of identical silicon nanoparticles with the radius R. Figure 3 shows the cross-sectional top-view of three different arrangements of these layers. Figure 3a, is a Simple Cubic (SC) arrangement where we assume that the cross-sectional location of particle centers in the upper layer resides on the particle centers in the lower layer. Figure 3b, is a Body-Centered Cubic (BCC) arrangement where the upper layer—shown inside the dashed square has a rectangular cross-section and—is shifted with a lattice vector $R\,\hat{a}_x + R\,\hat{a}_y$ with respect to its below layer. Finally in Fig. 3c SNPs form a Hexagonal Close-Packed (HCP) array wherein SNPs in the upper layer have the same pattern and location as the below layer. In terms of the absorber density we have HCP > BCC > SC. The reflection from these structures are compared for $R = 100\,\mathrm{nm}$ in Fig. 3d. As can be seen, the reflection from the first and third arrangements have very similar behavior at shorter wavelengths; at higher wavelengths, the HCP structure has an improved absorption due to its new resonances. The reflection corresponding to the BCC lattice presents a fairly fluctuating behavior; while the reflection is reduced in several wavelength intervals, between 680 and 780 nm, it is increased. The total reflection from these structures is also shown in the figure. Moreover, the photocurrent produced by each one is obtained via^29^

1 $$\begin{aligned} J_\mathrm{ph} = \frac{e}{hc}\int ^{\lambda = 1100 \mathrm{nm}}_{\lambda = 300 \mathrm{nm}} S(\lambda )A(\lambda )\lambda d\lambda , \end{aligned}$$

where *c* is the speed of light, *e* is the electron charge, *h* is Planck’s constant, and $S(\lambda )$ is AM 1.5G solar spectrum^30^. The reflection values show that the second form of periodicity—that much more resembles a quasi-random distribution—can generate larger $J_\mathrm{ph}$. Despite this, we consider the worst scenario (i.e. the first form of periodicity) when simulating the electrical behavior of these structures in the following sections.

### The absorption according to the incident angle

**Figure 4:**
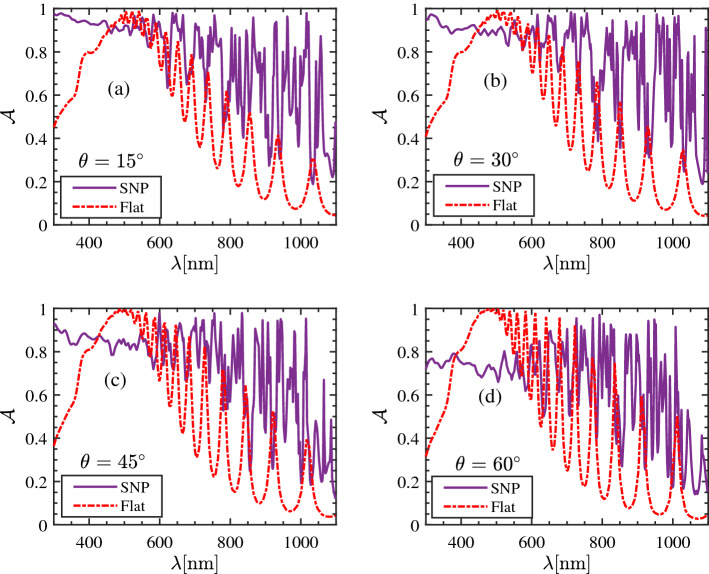
Impact of the angle of incidence on total absorption of a SNP structure and a flat silicon layer. SNP unit cell is composed of two particles in a dielectric medium with refractive index of 1.8 and thickness of 50 nm on top, all placed on a silver layer (NP radius = 300 nm). The angles of incidents are (**a**) $15^\circ$, (**b**) $30^\circ$, (**c**) $45^\circ$ and (**d**) $60^\circ$.

We now investigate the dependency of Fig. 1a to the angles of the incidence. Likewise the previous section, we concentrate on a SNP structure composed of a two-layer absorber covered by a dielectric medium with the thickness of $50\, \mathrm{nm}$ and refractive index of 1.8, and compute the absorption coefficient *A* at four different angles of incident $\theta = 15^\circ$, $30^\circ$, $45^\circ$ and $60^\circ$ through the interested spectrum. Results are shown in Fig. 4a–d. In addition, the absorption of the SNP structure at each incident angle is compared with the structure in Fig. 1b with a silicon layer of equal thickness. As can be seen, the absorption of the multilayered particles does not seriously change at $\lambda > 600\, \mathrm{nm}$. In contrast, at shorter wavelengths, $\mathcal {A}$ experiences a noticeable reduction, particularly at $\theta = 45^\circ$ and $60^\circ$. This can be explained using the ray-optic point of view; as the incident beam hits obliquely on the top particles, only a little portion of top the surface belonging to the upper particles has this chance to interact with light. This is in contrast to the normal incidence, where the whole surface receives light. In a similar way, one can argue about the negligible angle-dependence of the flat structure through the whole spectrum. However, by computing the photocurrent due to the absorption in both structures, it reveals that at the angle $\theta = 60^\circ$ the SNP structure still provides higher $J_\mathrm{ph}$ ($27.8 \,\mathrm{mA} \,\mathrm{cm}^-2$) than the flat absorber ($21.6\, \mathrm{mA}\,\mathrm{cm}^-2$). This again indicates that despite the reduction in the absorption efficiency, a SNP absorber is optically preferred to a flat one.

**Figure 5:**
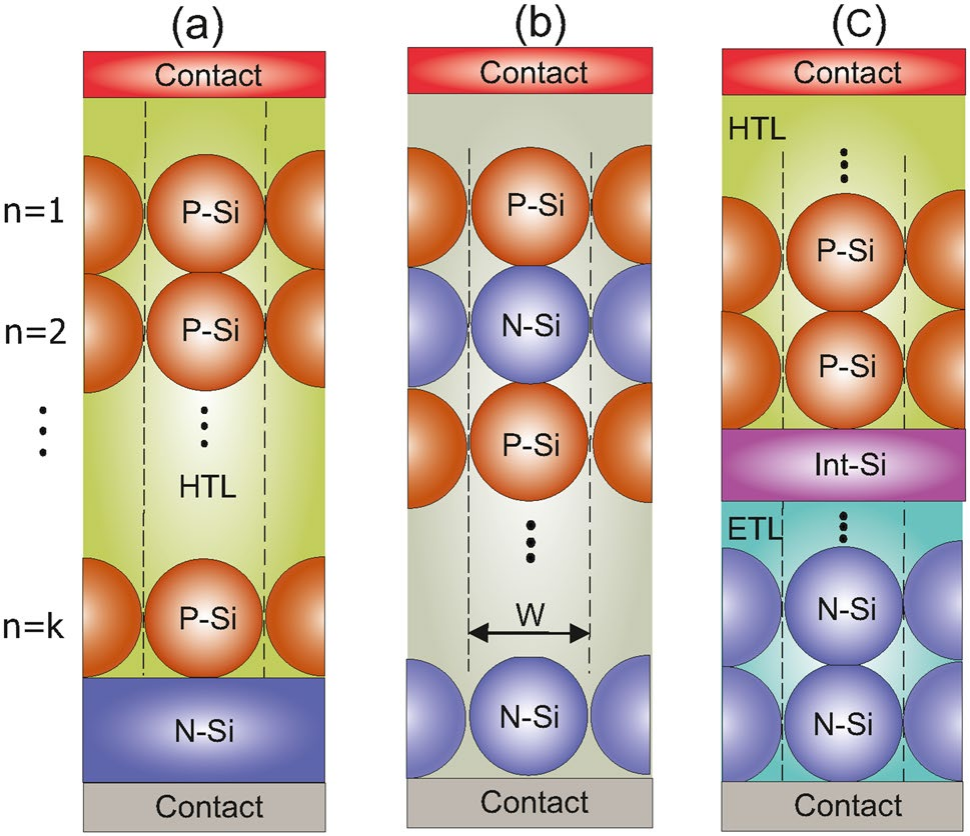
The schematics of Si SNP structures covered in this work: (**a**) structure A: cell with P-Si NPs, (**b**) structure B: cell with P-Si NPs and N-Si NPs without intermediate Int-Si layer, and (**c**) structure C: cell with P-Si NPs, N-Si NPs, and intermediate Int-Si layer.

## Solar cell performances with different cell configurations

Various configurations of SNP structures can be considered to operate as a solar cell. These structures can be categorized according to the particle size, type of distribution (i.e. periodic or random) and the operating mechanism designed for the cell. In terms of dimensions, we concentrate on SNPs with submicron dimensions. Also, cells based on silicon quantum dots are not considered here because poor carrier conductivity is still a serious drawback toward using them in photovoltaics^31^. Within this dimension interval (i.e. SNPs of a few hundreds of nanometer dimensions) the particle band gap remains unchanged. In terms of cell mechanism, we focus on configurations having p–n junctions. Depending on materials used as a host of nanoparticles there exist other quasi-p–n schemes that may be used to form a real SNP cell. They will be discussed in the next sections.

Figure 5 shows the cross-section of three cell configurations where particles have formed several layers. We here assume that particles have spherical shape so as to propose our main ideas. Figure 5a shows a unit cell composed of several layers of doped silicon particles inside a dielectric medium. The p-type silicon (P-Si) particles are placed above a n-type silicon (N-Si) layer to form a p–n junction. In this sense, the carrier transport toward contacts occurs through particle interfaces with adjacent particles or with the N-SI layer. The medium surrounding the nanoparticles can be assumed air. However, for the stability reasons, this is not a practical idea. Instead, assuming P-Si particles, then their surrounding medium can be a hole transport layer (HTL). This structure, henceforth called Structure A is a generalized form of the ultrathin structure proposed in^32^. The second structure (Structure B) shown in Fig. 5b is composed of merely multi-layers of SNPs that form a multi p–n junction cell inside a dielectric medium. Each layer has particles with a doping that is opposite to its adjacent layers. The main concern about this configuration is that particles in upper layers may diffuse into their below layers and hence, disrupt the expected carrier separation and transporting toward cell contacts. An alternative way to realize a structure with particles of various doping, is the configuration shown in Fig. 5c (Structure C). As seen, particle layers of different doping are separated with a thin interlayer medium. This layer can be of intrinsic silicon. In practice, this ultrathin layer may be realized using even smaller silicon nanoparticles. The P(N)-Si particles in this case are surrounded by a hole (electron) transport medium. A disadvantage of this structure is that particles below the intermediate layer do not effectively contribute in light confinement. However, the structure allows particles with various doping to form a cell structure. In the following, we first look at structure A and explore its optical and electrical parameters in a case study. Next, we look at the structure C and perform similar analysis to extract its electrical performance.

### Structure A

**Figure 6:**
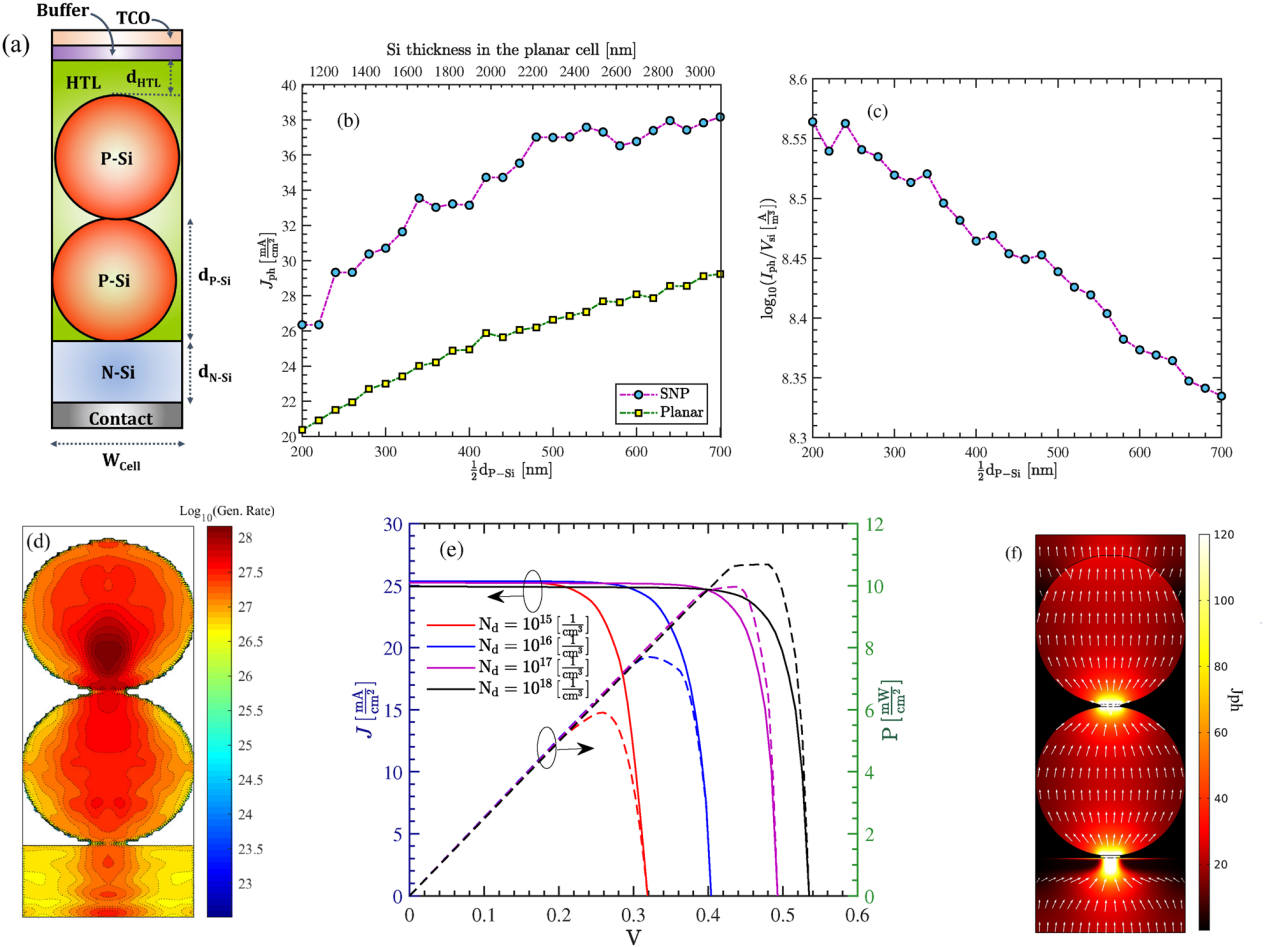
(**a**) Structure A with two P-Si nanoparticles. (**b**) The photocurrent in (**a**), when compared with a planar cell of identical thickness, as a function of particle size/absorber thickness. (**c**) The generated current per volume of the absorber for (**a**), as a function of particle radius. (**d**) The distribution of the generation rate in the cross-section of the unit cell in a logarithmic representation (unit is in $\mathrm{\mathrm m}^{-3}$). (**e**) J–V characteristics of the cell in (**a**), as a function of doping level of the silicon layer. (**f**) The total current density at V = 0.41 v in the unit of $\,\mathrm{mA} \,\mathrm{cm}^-2$. Arrows show the direction and distribution of the normalized current density.

In this section, we numerically study structure A shown in Fig. 6a. We will also compare the cell performances between the SNP cells with identical and non-identical P-Si NPs. As shown in Fig. 6a the simulated cell is composed of two identical P-Si nanoparticles immersed in a HTL. Regarding the top contact, a transparent conductive oxide (TCO) with the refractive index of 1.8 is considered. We have also assumed that there exists an identical contact area between the nanoparticles. The HTL is assumed to be the organic polymer PEDOT:PSS in this case study, and has covered the particles with the thickness $\mathrm{d}_\mathrm{HTL}$. A very thin buffer layer—with similar index of refraction as the TCO—is also included between the HTL and TCO, to protect the HTL from parasitic absorption. We note that solutions composed of silicon nanocrystals in polymers have been recently demonstrated for cheap and flexible optoelectronic applications^33^. Main material specifications and geometrical parameters of our case study are brought in Table 1.

Considering the dimensions given in the table, Fig. 6b compares the photocurrent $J_\mathrm{ph}$ generated by the cell as a function of particle dimensions (All particles have identical size). These results are compared with the produced current a conventional silicon cell having identical thickness to the particle-based structure (i.e. Thickness = $\mathrm{d}_\mathrm{HTL}+\mathrm{d}_\mathrm{N-Si}+\mathrm{d}_\mathrm{P-Si}$). As can be seen, the proposed cell offers approximately 30% higher photocurrent in comparison to the flat cell. Thus, despite having less volume of absorber, the particle-based cell acts highly efficient for light trapping. As the particle dimension increases, the photocurrent takes naturally higher values. However, if we draw the ratio of photocurrent to the silicon volume used (see Fig. 6c), we observe a downward trend with increasing the particle dimension, that indicates cell is becoming less efficient in terms of the absorber material consumed. Figure 6d shows the distribution of the carrier generation rate—in a logarithmic scale—of the cell in its cross-section. The generation rate is higher in the upper silicon particle; it is also highly concentrated in the bulk of the particles rather than the their boundaries. The current/power-voltage characteristics of the cell is obtained for various dopings of silicon layer in Fig. 6e. As can be seen, by increasing the doping value, the open circuit voltage also improves.This is because the dark saturation current density is decreased by increasing doping. This improves the cell efficiency from 5.8% for $\mathrm{N}_\mathrm{d} = 10^{15} \,\mathrm{cm}^{-3}$, to about 11% for $\mathrm{N}_\mathrm{d} = 10^{18} \,\mathrm{cm}^{-3}$. Despite this, the short circuit current remains almost unchanged with doping variation. This is because that the structure is basically a diffusion device. That is, the dominant carrier transport is the diffusion current, in which minority carriers in the P-Si nanoparticles (i.e. photo-generated electrons) move down to the N-Si. By increasing the doping density of the N-Si layer, the depletion width becomes narrower. However, the drift current is not a dominant factor to influence the total current. That is, not only the doping concentration of the P-Si nanoparticles but the photo-generated electrons in particles are remained unchanged. Thus, the short circuit current does not change noticeably.

Figure 6f shows the distribution of total current density at V = 0.41 v when $\mathrm{N}_\mathrm{d} = 10^{18}\, \mathrm{cm}^{-3}$. In addition, arrows show the direction of the normalized current density in the structure cross-section. As can be seen, in particle contacts, the current has critically high densities—up to 120 $\,\mathrm{mA} \,\mathrm{cm}^-2$; at the top surface of the upper particle the current is much widely distributed with slightly higher values around highest point.

**Figure 7:**
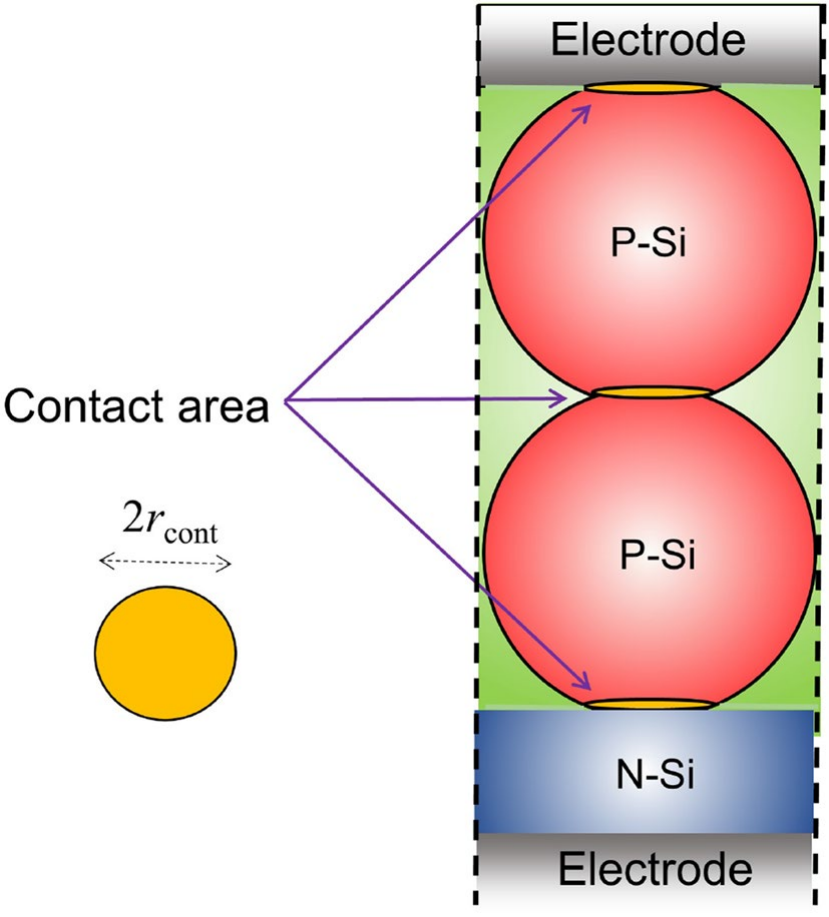
Structure A composed of two SNPs with imperfect shapes. A small circular interface on top and below of each nanoparticle is defined with the radius $r_\mathrm{cont}$.

The commercially produced nanoparticles are hardly pure spherical^34^. More often, they are characterized besed on their average physical size (APS). As a result, contacts between particles is an area rather than a single point. To consider this in modelings, we assume that the upper and below area of each particle are cropped. This forms a circular contact area as shown in Fig. 7. We note that in extracting the I–V characteristics we have assumed that the contact area on upper and lower part of the nanoparticles has the radius $r_\mathrm{cont.} = 60\,\mathrm{nm}$. As we reduce this interface, the cell efficiency will be reduced. Table 2 shows the variation of the efficiency, short circuit and open circuit voltage as a function of $r_\mathrm{cont.}$.

**Table 1:** Geometrical specifications and material properties of the structure shown in Fig. 6a.

Geometrical parameters		Silicon		PEDOT:PSS	
$\mathrm{d}_{\mathrm{N-Si}}$	300 (nm)	DC Permittivity	11.7	Permittivity	3.8
$\mathrm{d}_{\mathrm{P-Si}}$	600 (nm)	$n+i\kappa$	From Palik^35^	Hole mobility	$5 \times 10^{{ - 4}} {\kern 1pt} {\kern 1pt} {\text{cm}}^{2} {\kern 1pt} {\text{V}}^{{ - {\text{1}}}}$
$\mathrm{d}_{\mathrm{HTL}}$	50 (nm)	Sur. Rec. Vel.	Neglected	Electron affinity	5.1(ev)
$\mathrm{W}_{\mathrm{cell}}$	600 (nm)	P-Si doping	$\mathrm 10^{17}\, {\rm{cm}}^{-3}$	Bandgap	1.65 (ev)
$r_\mathrm{cont.}$	60 ($\mathrm{nm}$)	SRH, Rad. Auger Rec.	From^36^	Electron concentration	$2\times 10^{18}\, \mathrm{cm}^{-3}$

**Table 2:** Effect of contact area on electrical parameters of the cell shown in Fig. 6a.

$R_\mathrm{cont.}\,(\mathrm{nm})$	$V_\mathrm{oc}$ (V)	$J_{{{\text{sc}}}} \;{\text{mA}}\;{\text{cm}}^{{ - 2}}$	$\eta \%$
60	0.492	25.24	9.96
40	0.473	12.26	4.36
20	0.472	11.41	4.13

The doping of the silicon layer is assumed to be $10^{17} \,\mathrm{cm}^{-3}$.

**Figure 8:**
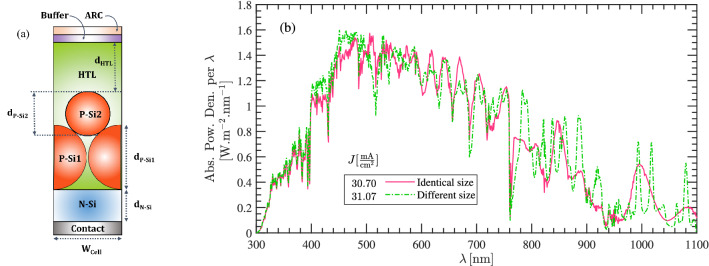
(**a**) Schematics of a cell with non-identical dimensions. (**b**) Impact of particle size on the absorbed power and $J_\mathrm{ph}$ of the cell shown in and (**a**) and that in Fig. 6a. The dimensions of the unit cell (**a**) are $\mathrm{d}_{\mathrm{p-Si1}} = 700\,\mathrm{nm}$, $\mathrm{d}_{\mathrm{p-Si2}} = 500\,\mathrm{nm}$, $\mathrm{d}_{\mathrm{N-Si}} = 300\,\mathrm{nm}$ and $\mathrm{d}_{\mathrm{HTL}} \approx 150\,\mathrm{nm}$. The dimensions of the identical structure is brought in Table 1.

Finally, we mention that the above calculations are obtained with the assumption that silicon nanoparticles have identical size. In practice, particles in a SNP cell may have different size. However, provided that the total thickness of the consumed silicon is unchanged, the overall absorption does not change significantly and hence, the results of Fig. 6a are valid. We look into the impact of using particles with different size in Fig. 8b. For this, consider Fig. 8a with its given dimensions in the caption. We compare the absorbed power density and the photocurrent with the structure of Fig. 6a with dimensions listed in Table 1. As can be seen, the absorbed power in the cell with non-identical size varies around the curve of the cell with identical size. Also, the photo-generated currents of the two structure are very close together.

**Figure 9:**
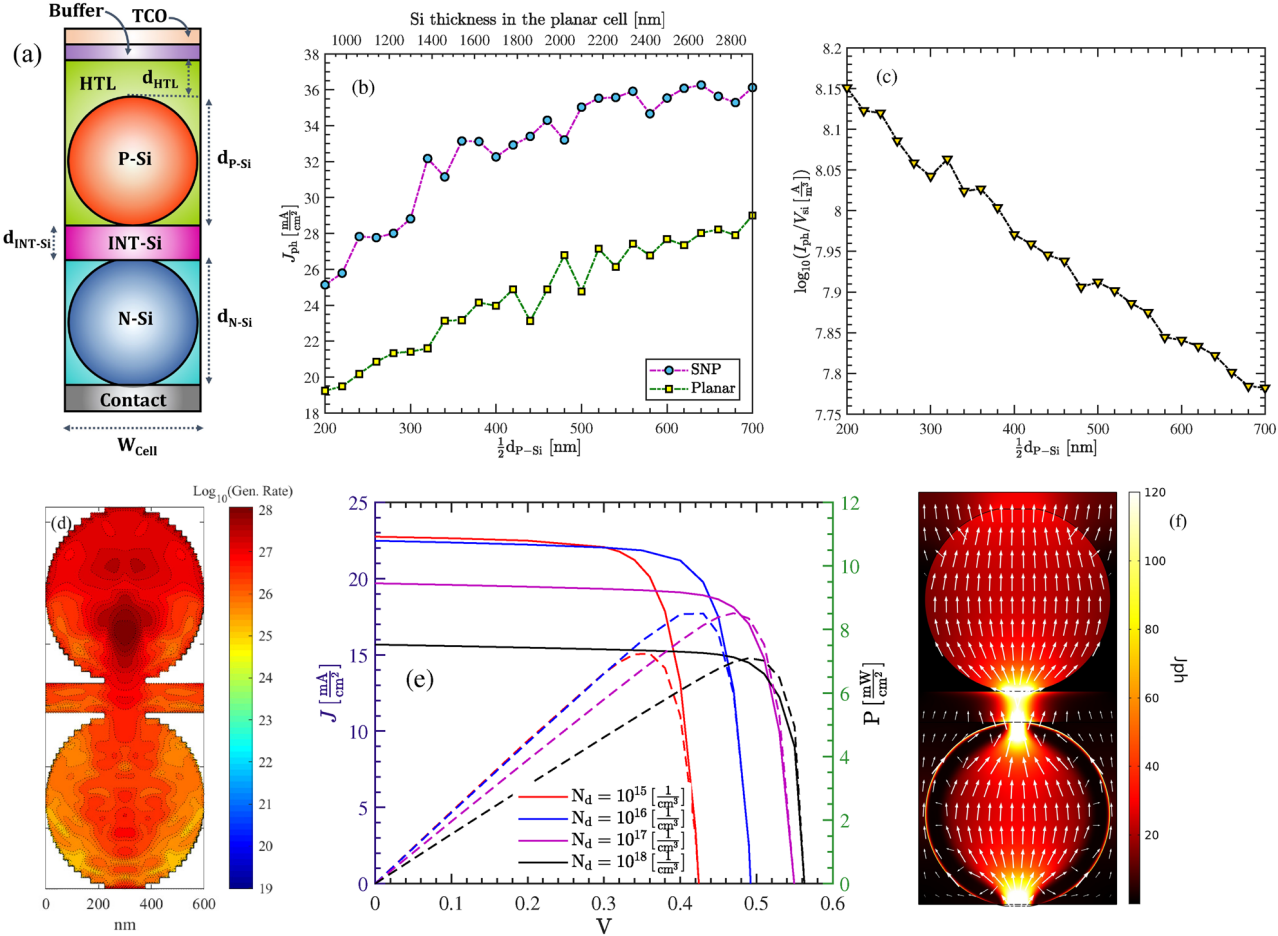
Example of structure C, composed of a P-Si and N-Si nanoparticles inside a HTL and ETL material, respectively. (**b**) shows the $J_\mathrm{ph}$ in structure shown in (**a**), and a planar cell of identical thickness, as a function of particle size/absorber thickness. (**c**) The generated current per volume of the absorber for the cell shown in (**a**), as a function of particle radius(i.e. $\frac{1}{2}\mathrm{d}_{\mathrm{P-Si}}$). (**d**) The distribution of the generation rate in the cross-section of the unit cell in a logarithmic representation (unit is in $\mathrm{m}^{-3}$). (**e**) J–V characteristics of the cell in (**a**), as a function of doping level of the silicon layer. (**f**) The total current density at V = 0.41 v in the unit of $\;{\text{mA}}\;{\text{cm}}^- 2$. Arrows show the direction and distribution of the normalized current density.

### Structure C

The next structure in our studies is an example of Structure C shown in Fig. 5c wherein, two nanoparticles with various doping are deposited on the upper and lower sides of an intrinsic silicon layer. The cell is shown in Fig. 9a and the geometrical parameters together with the electron transport layer(ETL) used in simulations are listed in Table 3. Note that the HTL is similar to the structure illustrated in the previous section. Figure 9b shows the photocurrent generated in the cell structure as a function of silicon particle size; the results are also compared with a conventional cell of the same thickness. A similar behavior to the previous case study can be seen for both cell structures (i.e. particle-based cell and conventional one). In terms of current produced per volume of the unit cell, Fig. 9c shows that smaller particles are more efficient despite generating lower levels of photocurrent. The generation rate of the unit cell over the cross section in Fig. 9d shows that most carriers are generated at upper nanoparticle and the generation rate reaches $10^{28}\frac{1}{\mathrm{s\, cm}^3}$ around the particle center. We have also looked at the I–V characteristic of the structure in Fig. 9e; we assumed that doping of the P-Si particle is $10^{17}\, \mathrm{cm}^{-3}$. Then, for various doping concentration of the N-Si particle, I–V graphs are obtained. In contrast to the structure A in the previous section, here, the short circuit current is significantly influenced by doping; At higher dopings, the short circuit current is reduced to $15.9 \;{\text{mA}}\;{\text{cm}}^{{ - 2}}$. In contrast to the structure A, here is only a single layer of P-Si nanoparticle. The below particle layer is N-Si, and an intrinsic layer is sandwiched between the particles. The device is therefore, like a PIN diode. The depletion region is now large and the drift current becomes a dominant mechanism. By increasing the doping in N-Si nanoparticle, the width of depletion region is reduced, hence fewer photo-carriers are generated inside that region. As a result, the drift current—which is the dominant one—is reduced by increasing the doping concentration. Although the open circuit voltage is increased by doping, the total power conversion efficiency is reduced and reaches 7%. Figure 9f shows the distribution of total current density at V = 0.41 v when $\mathrm{N}_\mathrm{d} = 10^{18}\, \mathrm{cm}^{-3}$. Likewise Fig. 6f, arrows show the direction of the normalized current density in the structure cross-section. As can be seen, the current density is strongly concentrated at the center of the below particle; this is because much weaker light hits to that particle and the carrier transport is limited to particle contacts; at the top surface of the upper particle, current is again much widely distributed over the particle surface.

**Table 3:** Geometrical specifications and material properties of the structure shown in Fig. 9a.

Geometrical parameters		SnO_2_	
$\mathrm{d}_{\mathrm{N-Si}}$	300 (nm)	DC Permittivity	11.7
$\mathrm{d}_{\mathrm{P-Si}}$	600 (nm)	$n+i\kappa$	From^35^
$\mathrm{d}_{\mathrm{HTL}}$	50 (nm)	Sur. Rec. Vel.	Vary
$\mathrm{W}_{\mathrm{cell}}$	600 (nm)	P-Si Doping	$10^{17}\,\mathrm{cm}^{-3}$
$r_\mathrm{cont.}$	60 ($\rm nm$)	SRH, Rad. Auger Rec.	From^36^

**Table 4:** Comparison between the key parameters of thin film silicon solar cells and structure A of this study.

Cell structure	Thickness ($\upmu \mathrm{m}$)	$\mathrm{J}_{\mathrm{sc}}\,(\;{\text{mA}}\;{\text{cm}}^{{ - 2}} )$	$\mathrm{V}_{\mathrm{oc}}\,(\mathrm{V})$	Efficiency%	References
Nanowire	8	16.82	0.559	5.30	^37^
Nanowire	2	28.4	0.52	9.9	^38^
Nanopyramid	0.7 + Si subs.	17.19	0.54	7.12	^39^
Inverted pyramid	2.75	25.3	0.44	5	^40^
Nanocone	10	31	0.5	9.62	^41^
Nanocone	6.8	19.1	0.552	6.2	^2^
Si layer with silica nanosphere	0.7	10.37	0.993	8.55	^42^
This work	0.95	25.24	0.492	9.96	

### Impact of $\mathrm SiO_2$ NP

**Figure 10:**
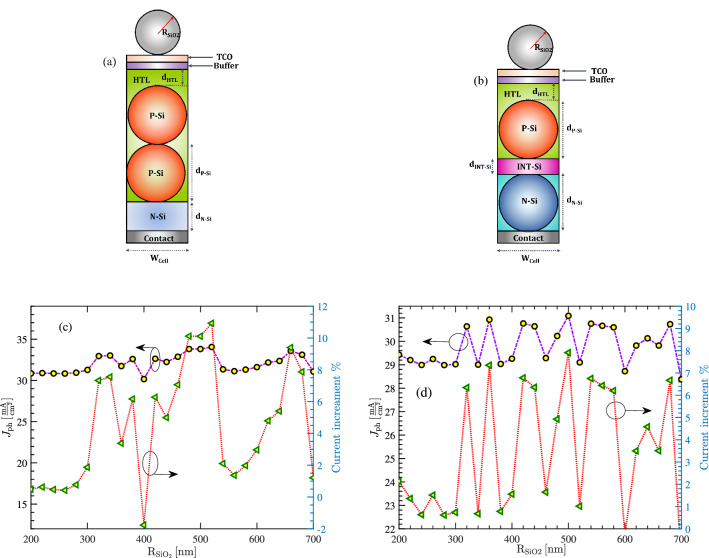
(**a**,**b**) The unit cell of the structures A and C, respectively when $\mathrm{SiO}_2$ nanoparticles are added on the top. (**c**,**d**) The total $J_\mathrm{ph}$ and current enhancement in the presence of silica nanoparticles of various dimensions, in (**a**,**b**), respectively. (SNP radius = 300 nm).

Although the texturing nature of nanoparticles is an effective mean to enhance light trapping in the proposed cell structures, existing a contact layer can slightly reduce this benefit. This is due to the flat structure of these layers as shown in Figs. 6a and 9a. One idea to further improve light trapping is to use $\mathrm{SiO}_2$ nanoparticles on top of a cell; this has been demonstrated in^43^. Figure 10a,b show the schematic of the structures 1 and 2 when $\mathrm{SiO}_2$ nanoparticles are included. In addition, Fig. 10c,d show the total photocurrent and the current enhancement as a function of $\mathrm{SiO}_2$ particle size in structures shown in Figs. 6a and 9a, respectively. As can be seen, the presence of $\mathrm{SiO}_2$ nanoparticles can enhance the photocurrent in both structures; smaller particles with—with the radius $\mathrm{R}_\mathrm{SiO2} < 300\,\mathrm{nm}$ lead to negligible current enhancement in both cells. As we use larger particles, the current enhancement reaches above 10% at $\mathrm{R}_\mathrm{SiO2} = 500\,\mathrm{nm}$ in the first cell and 8% in the second structure. The sensitivity of enhancement factor to the particle size is due to the interaction of different Mie resonances in $\mathrm{SiO}_{2}$ and Si nanoparticles.

## Discussion

The cell configurations introduced in this study provide improved optical absorption in an inherently textured structure. Interestingly, these features even happen for configurations with random distributions of nanoparticles; this issue leads to a simplified way for fabrication of these ultrathin cells. The overall efficiencies determined for the multi-layer SNP cells show a competing results with those of a nanowire cell. Table 4 shows the reported electric parameters of several nanowire solar cells. The table indicates that despite a rather low open circuit voltage—which is also common in nanowire cells—the expected efficiency of the SNP cell is remarkable.

While realizing a p–n junction was the basis for configuring and analysis of the described cells, by using hole transport polymers such PEDOT:PSS in contact with N-Si in Fig. 6a, a hybrid organic-inorganic junction is also formed. It is demonstrated that such these contacts act almost like a quasi p–n junction by Jäckle et al.^44^. Based on the free carrier movement in a simple PEDOT:PSS/N-Si junction, the generated carriers in our case studies is expected to constructively contribute in the overall current density. Another issue is about the effect of crystallinity of the silicon nanoparticle. We only considered crystalline silicon particles in the simulations. Cells based on amorphous silicon nanoparticles present weak carrier mobilities despite having high absorption. Therefore, they provide poor conversion efficiencies^33^.

**Figure 11:**
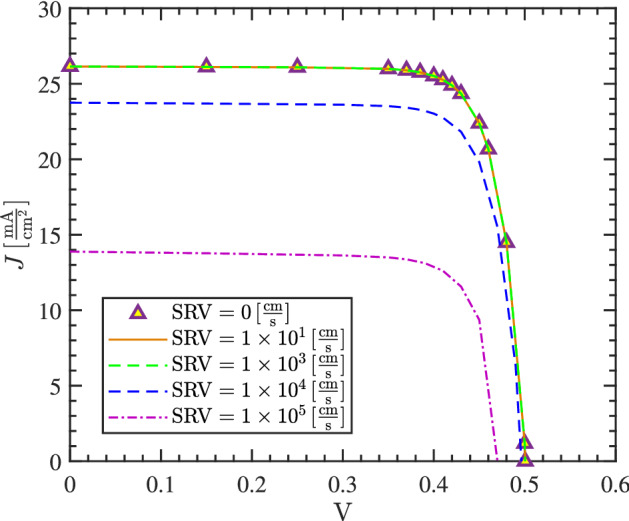
Impact of SRV on the J–V plot of the structure A. The dimensions and material properties of the cell are shown in Table 1.

A critical challenge on proper functionality of the proposed structures is the impact of recombination. Although we have included the conventional loss mechanisms in the models, the effect of surface recombination was neglected. In the following, we separately investigate impact of this loss by adding a recombination rate on particles as well as the front side of the silicon layer in the structure A. Figure 11 shows the J–V plot of the cell assuming the dimensions and material properties listed in Table 1. The particles doping are assumed to be $10^{17} \,\mathrm{cm}^{-3}$ and the silicon layer is assumed to have the doping $10^{17} \,\mathrm{cm}^{-3}$. As can be seen, by increasing the recombination velocity up to $10^{3}\,\mathrm{cm\,s}^{-1}$, the J–V graph remains almost unchanged. At higher recombination velocities, both open circuit voltage and short circuit current are reduced. Due to that, the cell efficiency drops to 5.6% for the surface recombination velocity $\mathrm{SRV} = 10^{5}\,\mathrm{cm\,s}^{-1}$. The graph shows that only at high surface recombination rates, efficiency is affected. This is because the carrier generation is mostly concentrated in the bulk of nanoparticles, away from the particle surface; the surface recombination is therefore effective on particle upper and lower areas where carrier transport happens.

We assumed that the silicon bandgap is unchanged by doping. At high levels of doping, bandgap narrowing appears which limits the increment of open circuit voltage^45^. In addition, we did not consider series resistance in the presented electrical calculations because the main purpose of this work was to propose the conceptual design of the structure without concentrating on a specific contact; Apparently, resistance—which is proportional to the contact material—slightly reduces the fill factor and hence, the cell efficiency. An inevitable consideration about the top contact is to choose materials which prevent diffusion of oxygen to the hole transport medium and silicon particles. This is because the emerging oxide layer around the nanoparticles can affect cell performance. Although thin oxide layers (say below 1 nm) can help in passivation of dangling bonds on the particle surface, further increase in the thickness prevents carrier transport between particles.

## Conclusion

In this paper, we proposed that multi-layer silicon nanoparticles of submicron dimensions can be deployed as the absorber of an ultrathin solar cell. We provided a parametric analysis to study the absorption behavior of the stack of these Mie scatterers and showed that the absorption efficiency of the structure is higher at lower number of layers and it approaches to absorption of a flat silicon layer for higher number of layers. We showed that in a dense distribution of silicon nanoparticles, their periodicity play a negligible role in absorption improvement. Several configurations were introduced to tailor these particles as a p–n junction cell. We finally investigated the electrical performance of selected case studies and found that the theoretical efficiency can reach about 11%, which is a promising value for such an ultrathin structure. Moreover, we showed that by including silica nanoparticles of proper size on top of the cell structure, one can enhance the photocurrent up to around 10%.

## Methods

In this paper, we numerically investigated the optical performance of the proposed solar cell with multi-layer silicon nano-spheres via full-wave simulation in CST. Due to the resonant nature of a SNP solar cell, we used the FDFD solver to achieve appropriate response accuracy; this solver also allows choosing the number of simulated frequencies in the interested interval. We applied periodic boundary conditions under normally(or obliquely, if needed) propagation of plane wave. The absorbed power inside the silicon parts, together with the corresponding generation rate were calculated. For the electric analysis, we then simulate the 3D cell structures in the Charge module of Lumerical. For this, each sphere was modeled as a stack of 3D polygons having thin thicknesses. The obtained generation rate is imported in the simulation. In addition, the material properties including permittivities, dopings and losses found in the literature were considered for silicon, silver and polymer parts.

## Data Availability

The datasets generated during and/or analysed during the current study are available from the corresponding author on reasonable request.
